# Does coffee protect against hepatocellular carcinoma?

**DOI:** 10.1038/sj.bjc.6600582

**Published:** 2002-10-21

**Authors:** S Gallus, M Bertuzzi, A Tavani, C Bosetti, E Negri, C La Vecchia, P Lagiou, D Trichopoulos

**Affiliations:** Istituto di Ricerche Farmacologiche ‘Mario Negri’, 20157 Milano, Italy; Istituto di Statistica Medica e Biometria, Università degli Studi di Milano, 20133 Milano, Italy; Department of Hygiene and Epidemiology, University of Athens Medical School, Goudi, Athens 115-27, Greece; Department of Epidemiology and Center for Cancer Prevention, Harvard School of Public Health, Boston, MA 02115, USA

**Keywords:** coffee, hepatocellular carcinoma, risk factors, case-control study

## Abstract

We analysed the relation between coffee consumption and hepatocellular carcinoma in two case-control studies conducted between 1984 and 1998 in Italy and Greece, including 834 cases and 1912 controls. Compared to non coffee drinkers, the multivariate odds ratio was 0.7 for drinkers of three or more cups per day.

*British Journal of Cancer* (2002) **87**, 956–959. doi:10.1038/sj.bjc.6600582 www.bjcancer.com

© 2002 Cancer Research UK

## 

The possible relation between coffee drinking and the risk of several cancers, particularly of cancers of the urinary bladder, pancreas and colorectum, has been widely investigated. These have shown a direct relation with bladder, an inverse one with colorectum, but no consistent association with other major sites, including liver cancer (Iarc, 1991; Tavani and La Vecchia, 2000).

Coffee drinking has been inversely related to the risk of liver cirrhosis in several studies (Klatsky and Armstrong, 1992; Klatsky *et al*, 1993; Corrao *et al*, 1994, 2001; Gallus *et al*, 2002). Although cirrhosis is a major correlate of hepatocellular carcinoma (Adami *et al*, 1992; La Vecchia *et al*, 1998, Kuper *et al*, 2000), the relation between coffee drinking and risk of hepatocellular carcinoma has been examined in only two studies which provided, however, no definite results. An Italian case-control study (La Vecchia *et al*, 1989), based on 151 cases with hepatocellular carcinoma, reported a multivariate odds ratio (OR) of 0.78 for drinkers of ⩾3 cups of coffee per day, compared to non coffee drinkers. In a Greek case-control study (Kuper *et al*, 2000), based on 333 cases, the age- and sex- adjusted OR was 0.7 for drinkers of ⩾20 cups per week compared to non drinkers.

To clarify the role of coffee drinking in hepatocellular cancer, we updated and re-analysed the Italian and Greek studies.

## MATERIALS AND METHODS

### Selection of cases and controls

The present data derived from two case-control studies of hepatocellular carcinoma. The first was conducted between 1984 and 1997, in a network of teaching and general hospitals in the Greater Milan area (La Vecchia *et al*, 1989, 1998). Cases were 501 subjects with incident, histologically confirmed hepatocellular carcinoma. Of these, 378 were males and 123 were females; the age range was 20–75 years, median age 60. Controls were 1552 patients with acute non neoplastic conditions, unrelated to long-term changes in diet or coffee drinking habit (i.e., gastritis or other chronic digestive tract disorders), from the same catchment areas as the cases, and admitted to the same hospitals. Of these, 1141 were males and 411 were females; the age range was 18–75 years, median age 56.

The second study was conducted between 1995 and 1998 in three teaching hospitals from Athens (Kuper *et al*, 2000). Cases were 333 subjects with incident hepatocellular carcinoma. Of these, 283 were males and 50 were females; the age range was 31–79 years, median age 65. Controls were 360 patients admitted to the same hospitals as the cases, for injuries, or eye, ear, nose or throat conditions. Of these, 298 were males and 62 were females; the age range was 24–79 years, median age 65.

Thus, a total of 834 cases of hepatocellular carcinoma and 1912 controls were considered in the present analysis.

### Data collection

Different structured questionnaires were utilised in the two studies but both collected information on socio-demographic characteristics, including education and occupation, a few indicator foods, lifetime tobacco smoking and alcohol drinking, and anthropometric measures. In the Italian questionnaire, a self-reported medical history was available, including information on hepatitis, liver cirrhosis and diabetes. In the Greek study, biological samples were obtained from cases and controls to test for markers of infection with hepatitis B and C viruses, while a self-reported medical history was available for diabetes and cirrhosis. With reference to coffee consumption, the Italian questionnaire included data on number of cups of coffee (mainly expresso and mocha) per day during the year before the onset of the index disease, and duration of the habit; the Greek study included three items for coffee consumption (Greek coffee, instant coffee and filtered coffee). For each item, the number of cups per week and the total duration of the habit (in years) were elicited. Total daily coffee consumption was computed summing up these three items.

### Data analysis

ORs and the corresponding 95% confidence intervals (CI) were derived from unconditional multiple logistic regression (Breslow and Day, 1980). All the equations included terms for study, quinquennia of age and sex. Additional models included terms for education, body mass index (BMI), tobacco smoking, alcohol drinking, diabetes and hepatitis.

## RESULTS

Table 1

**Table 1:**
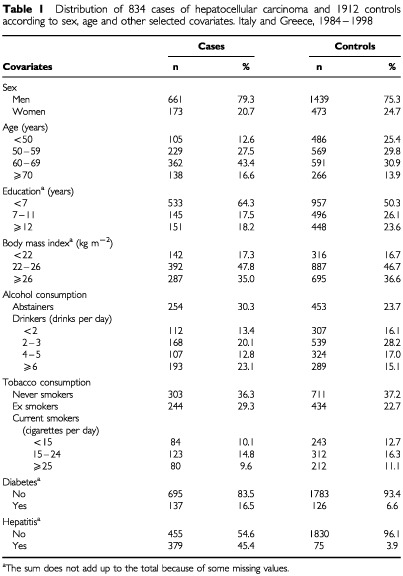
Distribution of 834 cases of hepatocellular carcinoma and 1912 controls according to sex, age and other selected covariates. Italy and Greece, 1984–1998

shows the distribution of hepatocellular carcioma cases and controls according to sex, age and selected other variables. Cases were more frequently less educated and heavy alcohol drinkers than controls. Moreover, they had more frequently a history of diabetes and hepatitis.

Table 2

**Table 2:**
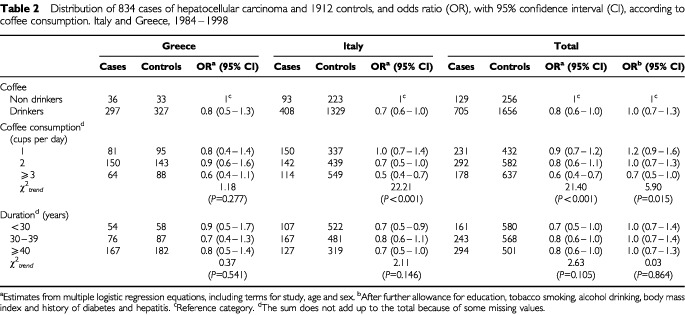
Distribution of 834 cases of hepatocellular carcinoma and 1912 controls, and odds ratio (OR), with 95% confidence interval (CI), according to coffee consumption. Italy and Greece, 1984–1998

shows the distribution of hepatocellular carcinoma cases and controls, and the corresponding ORs according to level and duration of regular coffee consumption. Compared to non coffee drinkers, the OR for coffee drinkers was 0.8 (95% CI: 0.6–1.0), and 0.6 (95% CI: 0.4–0.7) for drinkers of ⩾3 cups per day (*P* for trend <0.001). The inverse relation was consistent in the Italian and Greek datasets. After allowance for alcohol drinking, tobacco smoking and other potential confounding factors, these figures became 1.0 and 0.7, and the trend in risk with number of cups per day was significant. No association emerged with duration of coffee drinking, the OR being 0.8 for 40 years or more, compared to non coffee drinkers. When the reference category was set to subjects drinking ⩽1 cup per day, the multivariate ORs were 0.9 (95% CI: 0.7–1.1) for 2 and 0.7 (95% CI: 0.5–0.9) for ⩾3 cups per day.

Table 3

**Table 3:**
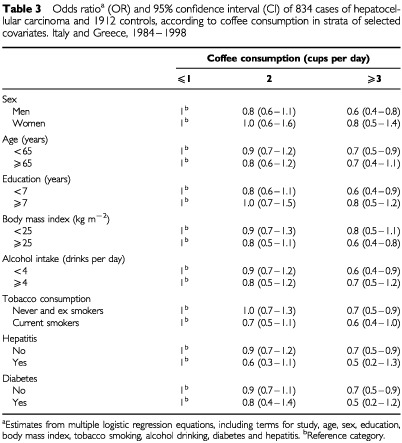
Odds ratio^a^ (OR) and 95% confidence interval (CI) of 834 cases of hepatocellular carcinoma and 1912 controls, according to coffee consumption in strata of selected covariates. Italy and Greece, 1984–1998

gives the multivariate ORs for subsequent levels of coffee consumption, as compared to ⩽1 cup per day, in all subjects and in strata of sex, age, education, BMI, alcohol and tobacco consumption, and history of diabetes and hepatitis. Although several of the ORs were not significant, in all strata ORs were below unity for heavy coffee drinkers (⩾3 cups per day), ranging between 0.5 and 0.8.

## DISCUSSION

This combined re-analysis suggests that coffee is inversely related to the risk of liver cancer. Coffee consumption has been inversely related to γ-glutamyltransferase (GGT) activity in studies from Italy, Finland, France, Japan and the United States (Kono *et al*, 1994; Tanaka *et al*, 1998; Casiglia *et al*, 1993; Poikolainen and Vartiainen, 1997; Aubin *et al*, 1998; Sharp and Benowitz, 1995; Sharp *et al*, 1999). GGT activity is a sensitive, but non-specific, indicator of several liver diseases, including liver cirrhosis and primary liver cancer (Penn and Worthington, 1983). Thus, it is plausible that coffee drinking has a real effect in reducing incidence of hepatocellular carcinoma, as suggested also by some experiments on rats and hamsters (Tanaka *et al*, 1990).

In several case-control studies, coffee consumption showed an inverse association with the incidence or diagnosis of liver cirrhosis, with significant trends in risk with dose and duration (Klatsky and Armstrong, 1992; Klatsky *et al*, 1993; Corrao *et al*, 1994, 2001; Gallus *et al*, 2002). Since liver cirrhosis is strongly related to the incidence of hepatocellular carcinoma (Adami *et al*, 1992; La Vecchia *et al*, 1998, Kuper *et al*, 2000), the apparent protective effect of coffee consumption on hepatocellular carcinogenesis may be due to its inverse relation with liver cirrhosis. A diagnosis of cirrhosis was not histologically determined in all cases, although most cases of liver cancer are likely to have some degree of cirrhosis. When the analysis was restricted to subjects who did not report history of cirrhosis, the multivariate OR for drinkers of ⩾3 cups of coffee, compared to non coffee drinkers, was 0.8 (95% CI: 0.6–1.0), suggesting that clinical history of cirrhosis cannot entirely account for this finding. Clinical history is however unlikely to reflect the real prevalence of cirrhosis among cases.

Weaknesses and strengths of the studies have been discussed elsewhere (Kuper *et al*, 2000; La Vecchia *et al*, 1989, 1998). Briefly, it is possible that coffee drinking in hospital controls differs from that of the general population (Rosenberg *et al*, 1981). However, we included in the control groups patients with diseases unrelated, or, at most, weakly positively related to the exposure under study. Information on coffee, moreover, was not materially influenced by hospital admission, and was satisfactorily reproducible (D'Avanzo *et al*, 1996). As for other possible sources of bias, the catchment areas of cases and controls were comparable, and participation was almost complete, since less than 5% of cases and controls refused the interview. Alcohol drinking and tobacco smoking (factors associated to the risk of hepatocellular carcinoma and coffee consumption) were allowed for in the analyses, together with education and other possible confounding factors. Additional allowance for selected indicator foods (vegetables and fruit) did not materially modify any of the results. Moreover, the association was observed in separate strata of tobacco smoking and alcohol drinking as well as age, sex and other major covariates of interest.

In conclusion, the present study confirms in a large dataset the hypothesis of an inverse association between coffee drinking and liver cancer. However, the interpretation of this association and the consequent inference on causality remains open.
